# Physical activity and social media addiction: a multi-mediation analysis

**DOI:** 10.3389/fpsyg.2026.1811877

**Published:** 2026-05-26

**Authors:** Cong Xue, Liping Deng, Zhiru Liang, Geng Li, Mingxiong Yang, Yanfei Wang, Jiale Wang, Bo Wang, Sishun Chen

**Affiliations:** 1 Jishou University, Jishou, China; 2 Hunan University of Medicine, Huaihua, China; 3 Department of Hepatobiliary Surgery, Xilingol League Central Hospital, Xilinhot, Inner Mongolia, China; 4 School of Psychology, Research Center for Exercise and Brain Science, Shanghai University of Sport, Shanghai, China; 5 Department of Science and Education, Xilingol League Central Hospital, Xilinhot, Inner Mongolia, China; 6 Xinjiang Normal University, Urumqi, China; 7 Baotou Medical College, Inner Mongolia University of Science and Technology, Baotou, China

**Keywords:** anxiety, college students, depression, physical activity, psychological resilience, social media addiction, stress

## Abstract

**Background and objectives:**

Physical activity levels are closely related to social media addiction among college students. However, the specific psychological mechanisms by which physical activity affects social media addiction remain to be further clarified. This study aims to introduce psychological resilience and negative emotional factors such as anxiety, depression, and stress to construct a chain-mediation model, systematically exploring the psychological mechanisms between physical activity and social media addiction among college students. The goal is to provide a more detailed theoretical explanation for understanding the protective effect of positive behaviors on problematic media use.

**Methods:**

In 2025, a self-report questionnaire survey was conducted among 2,033 university students (882 males and 1,151 females; mean age = 18.97 ± 1.13) using a convenience sampling approach. A cross-sectional design was employed to assess six core variables: anxiety, depression, stress, psychological resilience, physical activity, and social media addiction. Pearson correlation analysis was followed by mediation analysis using the PROCESS macro for SPSS.

**Results:**

Physical activity was significantly negatively associated with social media addiction (β = −0.117, *p* < 0.001), and this effect remained significant after including mediators (β = −0.068, *p* < 0.001). Physical activity was negatively correlated with anxiety (β = −0.054, *p* < 0.05) and depression (β = −0.077, *p* < 0.001), and positively correlated with psychological resilience (β = 0.132, *p* < 0.001). Psychological resilience, in turn, was negatively associated with anxiety (β = −0.271), stress (β = −0.178), depression (β = −0.259), and social media addiction (β = −0.080; all *p* < 0.001). Anxiety (β = 0.224), stress (β = 0.102), and depression (β = 0.114) were each positively related to social media addiction (*p* < 0.001).

**Conclusion:**

This study clarifies the psychological pathways linking Physical activity to social media addiction. The results suggest that psychological resilience, along with anxiety, depression, and stress, plays a key mediating role in this association. These findings underscore the importance of integrating positive lifestyle behaviors and core psychological resources into analytical frameworks, thereby providing valuable insights into the developmental pathways of social media addiction among university students and informing more targeted and effective intervention strategies.

## 1 Background

With the widespread adoption of social media platforms in higher education contexts, irrational patterns of use and the potential risk of addiction have increasingly become prominent concerns in research on university students' mental health (Andreassen and Pallesen, 2014). University students who engage in excessive and poorly controlled social media use and subsequently develop social media addiction (SMA) often experience difficulties in regulating usage duration and controlling motivational impulses, which may further intensify use dysregulation and addictive tendencies (Blackwell et al., 2017). SMA is commonly conceptualized as a form of behavioral addiction centered on social media engagement, and is typically characterized by salient urges to use, increased tolerance, withdrawal symptoms, and persistent negative consequences for academic functioning, social functioning, and psychological well-being (Tetik et al., 2025). A meta-analysis incorporating 51 original studies with a total sample of 35,520 participants reported a pooled prevalence of SMA of 18.4% among university students (Salari et al., 2025). Similarly, a cross-sectional survey conducted in Singapore involving 1,110 university students indicated that 29.5% of participants were at risk of SMA (Dalvi-Esfahani et al., 2019). In China, social media has become deeply embedded in the daily lives of university students and is widely used for academic communication, entertainment, information acquisition, decision-making, and mobile payment services. The total number of users has exceeded one billion, with individuals aged 15–29 accounting for more than 60% of this population (Office of the Central Cyberspace Affairs Commission, 2019). Nevertheless, excessive reliance on social media may result in reduced body satisfaction, impaired time management, and a variety of mental health problems (Andreassen et al., 2017). Therefore, identifying potential risk factors for problematic social media use is of substantial practical importance. Based on the existing literature, a comprehensive examination of the negative consequences of problematic social media use among university students not only facilitates a clearer understanding of the underlying psychological and behavioral mechanisms, but also provides valuable empirical evidence to inform mental health interventions in higher education, promote healthy media literacy, and support the development of rational guidelines for social media use.

### 1.1 Physical activity and social media addiction

The university stage represents a critical transitional period during which individuals shift from externally regulated behaviors to self-regulation. Within a relatively open environment, university students are required to independently manage academic responsibilities, social interactions, and daily activities. Consequently, lifestyle-related factors play a particularly important role in students' psychological adjustment and behavioral regulation. As a modifiable health behavior, physical activity (PA) is widely recognized for its potential protective effects, including the promotion of mental health, enhancement of self-regulatory capacity, and reduction of problematic behaviors (Huang et al., 2020). The WHO defines physical activity as skeletal-muscle-driven bodily movement requiring energy expenditure, primarily aimed at improving fitness and health (World Health Organization, 2024). For university students, regular engagement in PA not only contributes to improved physical functioning and enhanced immune capacity, but has also been shown to effectively alleviate negative emotional states such as anxiety (AN) and depression (DP) (Bull et al., 2020). However, at a global level, insufficient physical activity among adolescents and young adults has emerged as a prominent public health concern. A large-scale study covering 146 countries and approximately 1.6 million participants reported that 85% of females and 78% of males failed to meet recommended levels of PA (Guthold et al., 2020). Moreover, a cross-sectional study found that 76.88% of university students failed to achieve adequate levels of PA (Qin et al., 2024). According to the stress-buffering model, PA, as a form of active coping, helps reduce individuals' subjective perceptions of stressors while enhancing emotional regulation and self-control capacities, thereby decreasing the likelihood of addictive behaviors driven by avoidance or compensatory motivations (Gerber and Pühse, 2009). Existing research has consistently demonstrated a significant negative association between levels of PA and SMA, and that high levels of PA can reduce the occurrence of problematic media use (Xu and Tang, 2024). The above evidence suggests that PA plays a protective role in the behavioral regulation of college students. Based on this body of literature, the present study advances the hypothesis that PA is inversely linked to SMA among university students (H1).

### 1.2 Mediating effect of psychological resilience

Even at comparable levels of PA, substantial individual differences in social media use behaviors can be observed, suggesting that the influence of PA on SMA may not operate solely through direct pathways, but rather through internal psychological mechanisms. During the university period, individuals gradually transition from external regulation to self-regulation, with internal psychological resources playing an increasingly central role in behavioral control. Within this developmental context, psychological resilience (PR) contributes to the formation of coping strategies and behavioral patterns. PR is commonly defined as an individual's capacity to rapidly adapt to and recover from significant stress (ST) or crisis, thereby restoring psychological equilibrium (Wikipedia, 2025). This capacity reflects an individual's level of adaptation to psychological ST and functions to maintain mental health when confronted with disruptive or unexpected events, preventing prolonged negative outcomes (Herrman et al., 2011). A meta-analysis conducted in 2022 integrating data from 41 original studies across 18 countries, involving a total of 13,931 students, reported that the prevalence of low PR in student populations was 36% (Chua et al., 2023). Empirical evidence suggests that students who participate more frequently in physical activity tend to report elevated resilience levels (Lei et al., 2025). Furthermore, PR plays an important role in inhibiting tendencies toward SMA. According to compensatory internet use theory, when individuals are unable to obtain sufficient psychological resources to cope with stress in real-life contexts, they are more likely to engage in frequent social media use to alleviate negative emotions (NE), thereby increasing the risk of addiction (Kardefelt-Winther, 2014). In contrast, higher levels of PR enable university students to cope with stressors and emotional difficulties in more adaptive ways, reducing their reliance on external compensatory channels. Existing studies have consistently shown that PR is negatively associated with SMA and serves a buffering role across multiple risk pathways (Hou et al., 2019). In light of this theoretical and empirical foundation, the present study advances the hypothesis that PR functions as a mediating mechanism connecting PA and SMA among university students (H2).

### 1.3 Mediating effect of negative emotions

During the university stage, ST sources such as academic workload, interpersonal adaptation, and uncertainty regarding future development tend to converge. When individuals remain in a prolonged state of emotional regulation imbalance, they are highly susceptible to persistent symptoms of AN, ST, and DP. These negative emotional states are widely regarded as proximal psychological risk factors for SMA (Sun et al., 2024). The Dictionary of Psychology defines NE as emotional responses elicited when individuals face external threats, loss of interests, or goal frustration. Such emotions are typically accompanied by pronounced subjective discomfort and may exert disruptive effects on cognitive judgment, behavioral performance, and social functioning (VandenBos, 2007). A meta-analysis synthesizing data from 64 studies (*N* = 100,187) reported pooled prevalence rates of 33.6% for depressive symptoms and 30.0% for AN symptoms among university students (Li et al., 2022). In addition, a cross-sectional study of 688 university students found prevalence rates of 49% for depression, 75% for AN, and 73.26% for ST (Haruna et al., 2025). According to behavioral activation theory, one of the core characteristics of DP, AN, and ST is increased avoidance behavior and a reduction in exposure to positive reinforcement. PA can substantially increase individuals' contact with positive environmental stimuli, thereby disrupting the vicious cycle between avoidance and negative emotional states (Craft and Perna, 2004). Regular PA fosters pleasurable experiences and a sense of accomplishment, thereby alleviating DP, AN, and ST. University students face additional pressures—academic, familial, interpersonal, identity-related, and employment uncertainty—intensified by social competition (Núñez-Regueiro and Núñez-Regueiro, 2021). Empirical studies consistently demonstrate that PA is negatively associated with DP, AN, and ST (Mu et al., 2024), and these NE have been shown to be closely related to SMA (Foroughi et al., 2022; Zhao et al., 2023). According to the mood enhancement hypothesis, individuals experiencing negative emotional states are more inclined to engage in activities that can rapidly improve mood or divert attention in order to regulate current emotional discomfort (Bryant and Zillmann, 1984). University students affected by DP, AN, or ST may seek online interaction to fulfill emotional and interpersonal needs. However, concerns about unfavorable evaluation can discourage face-to-face communication (Kashdan et al., 2008). In contrast, social networking environments provide comparatively low-risk spaces that reduce exposure to direct scrutiny or critical feedback (Ando and Sakamoto, 2008). Consequently, individuals with psychosocial difficulties are more likely to express themselves through online social networks (Chen et al., 2024). Anticipation of rejection or criticism in offline settings can further motivate immersion in digital social contexts, reinforcing impulsive or excessive platform use and increasing susceptibility to SMA (Di Blasi et al., 2015; Shensa et al., 2017). In addition, ST has been found to be significantly positively associated with problematic social media use, indicating that university students experiencing higher levels of ST are more vulnerable to developing problematic media use behaviors (Fu et al., 2023). Drawing on this body of evidence, the present study formulates H3: AN, ST, and DP function as mediating mechanisms in the linkage between PA and SMA among university students.

### 1.4 The chain-mediating effect of psychological resilience and negative emotions

An expanding body of research further confirms the close interrelations among AN, ST, DP, and PR. Empirical findings indicate that individuals experiencing more severe depressive symptoms tend to demonstrate lower levels of PR. When confronted with academic or life setbacks, they often encounter greater difficulties in maintaining emotional stability and require extended periods to restore normal psychological functioning, thereby increasing the likelihood of emotional fluctuation and psychological exhaustion (Waugh and Koster, 2015). Under depressive conditions, individuals are more prone to negative self-evaluation and ruminative thinking patterns, which undermine adaptive coping processes and weaken the maintenance of PR (Hankin et al., 2015). In states of heightened AN, individuals typically remain persistently vigilant toward potential threats, with attentional resources continuously occupied. Such sustained cognitive load places the psychological system under considerable strain, thereby diminishing the capacity for flexible emotional regulation and effective ST coping (Ong et al., 2006). According to the psychological flexibility model, when individuals fail to appropriately adjust their responses to internal and external stimuli, PR correspondingly declines, and AN symptoms are more likely to persist and generalize (Kashdan and Rottenberg, 2010). Moreover, research suggests that individuals with lower levels of PR are more susceptible to pronounced increases in AN and DP under prolonged ST exposure (Kalisch et al., 2017). Based on the foregoing discussion, the present study hypothesizes that PR is negatively associated with DP, AN, and ST. Furthermore, PR, together with DP, AN, and ST, is expected to mediate the relationship between PA and SMA among university students (H4).

### 1.5 The current research

**Figure 1:**
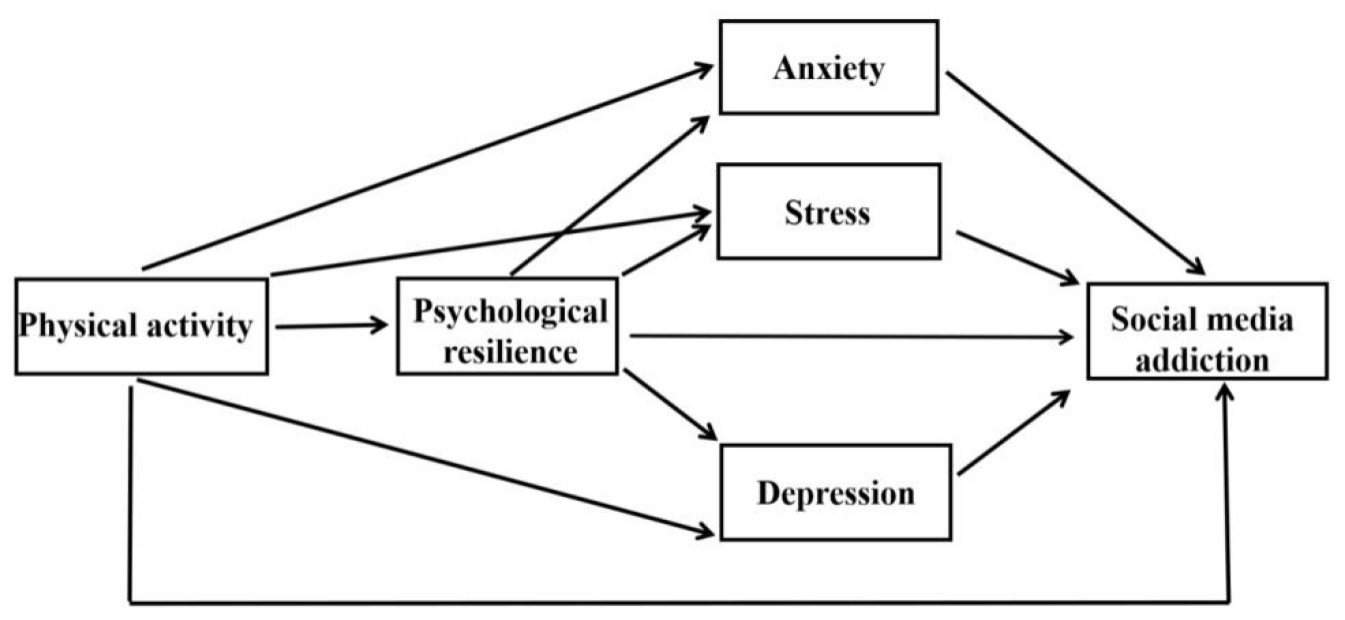
Chain hypothesized a mediation model.

Existing research has demonstrated close associations among PA, PR, negative emotional states, and SMA. However, most prior studies have primarily focused on bivariate relationships or examined single mediating pathways, with limited efforts to integrate multiple psychological mechanisms within a unified analytical framework. This limitation is particularly salient in the context of Chinese university students. Within the Chinese educational and sociocultural context, factors such as performance-oriented evaluation systems, high parental expectations, and intense social competition may contribute to more complex emotional responses and behavioral adjustment challenges among students. Despite this, relatively few studies have simultaneously incorporated PR and multiple NE (e.g., DP, AN, and ST) to systematically examine their joint and potentially sequential roles in the relationship between PA and SMA. Importantly, PR, as a core internal psychological resource, may play a foundational role in shaping how individuals cope with stress and regulate emotions, thereby influencing subsequent levels of depression, anxiety, and stress. These variables are unlikely to operate in isolation; rather, they may function in an ordered and interrelated manner. Specifically, PR may indirectly influence SMA through its effects on multiple dimensions of negative emotional states, forming a chain mediation process. However, empirical evidence supporting such a sequential mechanism remains limited, particularly within the Chinese cultural and familial context. To address this gap, the present study aims to develop and test a serial multiple mediation model incorporating PR, depression, anxiety, and stress, in order to provide a more comprehensive understanding of the mechanisms linking PA and SMA. By focusing on this integrative, multi-path framework, the study contributes to a deeper understanding of how psychological resources and emotional factors interact in the development of behavioral addiction among university students, while also offering empirical insights into the role of sociocultural context in shaping these processes (see Figure 1).

## 2 Methods

### 2.1 Participants

Data were collected during the autumn semester of 2025. A cluster random sampling approach was employed to recruit 2,194 university students from six provinces representing four major regions of China (North, Central, Southwest, and South China), in order to improve the geographic coverage and heterogeneity of the sample. During data screening, questionnaires were removed if they met any of the following conditions: (1) failure to correctly answer embedded attention-check (lie-detection) items designed to identify inattentive or random responses; (2) evidence of mechanical or patterned responding, such as repeatedly selecting the same response option across the majority of items or exhibiting repetitive wave-like answering patterns; and (3) excessively short completion times indicative of poor data quality. To ensure validity, questionnaires completed in less than 10 min were excluded from analysis.

After screening, 2,033 valid responses were retained, corresponding to an effective response rate of 92.65%. The final sample comprised 882 male and 1,151 female students, with a mean age of 18.97 years (M = 18.97, SD = 1.13). The distribution by academic year was as follows: first year (*n* = 905), second year (*n* = 1,010), third year (*n* = 111), and fourth year (*n* = 7).

Prior to the formal survey, the research team provided all participants with detailed information regarding the study objectives, questionnaire content, and the intended use of the data. The first page of the online questionnaire contained an electronic informed consent form and a brief description of the study, including information on data handling, complete confidentiality, and anonymization. Only participants who read and clicked “Agree” were allowed to proceed to the questionnaire; those who did not consent or who withdrew midway were fully respected, with no adverse consequences. The study adhered to the principles of the Declaration of Helsinki. Data were collected via online classroom distribution channels. Ethical approval was obtained from the institutional review board of the researchers' affiliated institution before data collection commenced.

### 2.2 Measures

Taking into account the academic stress faced by participants and the potential instability of online surveys, this study adopted concise measurement instruments to minimize interference with participants' daily academic activities and instructors' teaching schedules. The use of abbreviated instruments offers several advantages (Fisher et al., 2016; Allen et al., 2022), including reducing cognitive and time burdens, minimizing response bias, enhancing participant engagement, and simplifying data processing and analysis. By employing these streamlined measures, the present study achieved a balance between data quality and participant convenience, ultimately contributing to more accurate and meaningful research findings. In addition, single-item measurements are widely used in epidemiological and public health research, especially for assessing constructs that are general and subjective (such as overall PA level or ST). Previous studies have shown that single-item indicators are usually moderately correlated with multiple scales or objective measurements, have acceptable validity, and are highly practical in large-sample cross-sectional studies (Scott et al., 2015).

### 2.3 Measuring tools

#### 2.3.1 PA

PA were assessed using a single indicator measure. Participants determined their responses based on self-perceived exercise intensity (i.e., activity that caused sweating or shortness of breath) and a duration of at least 30 min. Responses ranged from 0 to 7 days [Centralia Elementary School District (n.d.)]. This measurement approach has been validated in previous research and has been shown to demonstrate good reliability and validity in university student samples (Waasdorp et al., 2019; Wang et al., 2025a; Jia et al., 2025).

#### 2.3.2 PR

PR was assessed using the Connor–Davidson Resilience Scale (CD-RISC) (Wang et al., 2010). The original 10-item version has been widely applied across cultural contexts. Previous studies have indicated that Items 1 and 5 adequately capture core resilience dimensions (Waddimba et al., 2022; Vaishnavi et al., 2007; Kuiper et al., 2019); however, based on item response theory analyses in a U.S. sample, Waddimba et al. recommended Items 2 and 9 as more reliable for a brief version (Waddimba et al., 2022). Considering these findings and the characteristics of the present sample, Items 2 and 9 were adopted. Responses were recorded on a 5-point Likert scale (1 = never to 5 = always), with summed scores ranging from 2 to 10. Higher scores indicate greater PR. In this sample, the two-item measure demonstrated satisfactory internal consistency (Cronbach's α = 0.869). Meanwhile, related studies have also confirmed that this measurement method has good reliability and validity among university students (Yan et al., 2026c).

#### 2.3.3 NE

AN and DP symptoms experienced over the past two weeks were measured using the GAD-2 (Byrd-Bredbenner et al., 2021) and PHQ-2 (Levis et al., 2020) respectively. Each instrument consists of two items, with total scores ranging from 2 to 8. In addition, ST was assessed using a single-item indicator (Elo et al., 2003). All items were rated on a 4-point Likert scale (1 = not at all; 4 = almost every day). In this study, the Cronbach's α coefficients were 0.852 and 0.863. Higher scores indicate greater severity of AN, DP, and ST symptoms. These scales have been widely used in clinical and non-clinical populations, including college students and young adults, and have shown satisfactory overall reliability (Thomée et al., 2011; Puolakanaho et al., 2023; Thomée et al., 2012; Merikanto et al., 2022; Härmä, 2006; Itzhaki et al., 2018; Yang et al., 2025; Yan et al., 2026a,b).

#### 2.3.4 SMA

Symptoms of SMA were measured using the Bergen Social Media Addiction Scale (BSMAS) (Andreassen et al., 2016). The BSMAS comprises six items reflecting six core components of addiction: salience, mood modification, tolerance, withdrawal, conflict, and relapse (Griffiths, 2005). Items were rated on a 5-point Likert scale (1 = very rarely to 5 = very often), yielding total scores from 6 to 30. The scale demonstrated strong internal consistency in this sample (Cronbach's α = 0.885, KMO = 0.879, *p* < 0.001).

### 2.4 Covariates

Gender, age, academic year, only-child status, and parental education level were included as covariates in the statistical models to control for potential confounding effects of demographic characteristics on the associations among the primary study variables, thereby improving the internal validity and interpretability of the findings.

### 2.5 Statistical analysis

All statistical procedures were performed using SPSS 29.0. Descriptive statistics and Pearson correlation coefficients were first computed for the primary study variables. The assumption of univariate normality for continuous variables was evaluated using the Shapiro–Wilk test, along with inspection of central tendency (M), dispersion (SD), skewness, and kurtosis indices. Distributions were regarded as approximately normal when the absolute value of skewness was below 2 and that of kurtosis was below 7 (Kim, 2013). Results suggested that the principal variables met normality criteria, supporting the application of parametric statistical techniques in subsequent analyses. Pearson correlation analyses were then conducted to examine relationships among the core variables. Prior to hypothesis testing, all principal variables were standardized.

To test the proposed hypotheses, the PROCESS macro (Model 81) for SPSS was employed to analyze the linkage between PA and SMA, as well as the mediating functions of PR and negative emotional states (AN, ST, and DP) (Hayes, 2018). Indirect effects were estimated via bootstrap resampling with 5,000 iterations, and 95% confidence intervals (CIs) were generated to determine statistical significance. All analyses adopted two-tailed testing with the significance threshold set at α = 0.05 (Berkovits et al., 2000). To further assess the robustness of the measurement structure, exploratory factor analysis was first conducted within a modeling framework to evaluate the factor loadings of the observed variables. Confirmatory factor analysis (CFA) was subsequently performed using AMOS 29.0 to assess the structural validity of the latent variable model.

Model fit was evaluated using multiple goodness-of-fit indices, including the chi-square to degrees of freedom ratio (χ^2^/df), root mean square error of approximation (RMSEA), goodness-of-fit index (GFI), comparative fit index (CFI), Tucker–Lewis index (TLI), normed fit index (NFI), and standardized root mean square residual (SRMR).

Model adequacy was interpreted according to the following criteria:

- 1.0 ≤ χ^2^/df ≤ 3.0 indicates good fit;
- 3.0 < χ^2^/df ≤ 5.0 indicates acceptable fit;
- 5.0 < χ^2^/df < 10.0 indicates substantial misfit;
- χ^2^/df ≥ 10.0 indicates poor fit.

For RMSEA, values below 0.05 indicate excellent fit, whereas values between 0.05 and 0.08 reflect acceptable fit. GFI, CFI, TLI, and NFI values greater than 0.90 were considered indicative of satisfactory model fit. For SRMR, values below 0.08 suggest adequate fit (Hu and Bentler, 1999; Lomax, 2012).

## 3 Results

### 3.1 Common method bias

To assess common method bias, Harman's single-factor test was conducted. Without rotation, four factors with eigenvalues >1 were extracted; the first factor explained 21.57% of the total variance, which is substantially below the commonly accepted threshold of 40%. These results indicate the absence of significant common method bias in the data.

Further examination through confirmatory factor analysis (CFA) conducted in AMOS 29.0 demonstrated satisfactory model fit: χ^2^/df = 2.04; CFI = 0.99; TLI = 0.98; NFI = 0.99; RMSEA = 0.04 (90% CI: 0.04–0.06); SRMR = 0.04. All standardized factor loadings were above 0.50 and reached statistical significance, supporting the adequacy of the measurement structure. This indicates a strong correlation between the observed variables and their corresponding latent factors, thus further validating the scale's good construct validity.

### 3.2 Descriptive analysis

Table 1 presents descriptive statistics and comparisons across gender, only-child status, and academic year. Significant gender differences were observed in PA, with male students reporting higher levels of PA compared to female students. Significant differences across academic years were identified for PA, PR, ST, DP, AN, and SMA. Specifically, freshman exhibited the highest levels of PR; sophomore reported the highest levels of DP and AN; junior demonstrated the highest levels of PA; and senior showed comparatively higher levels of ST and SMA relative to students in other academic years.

**Table 1:** Describes the analysis.

Variables		PA		PR		ST		DP		AN		SMA	
		Mean	Sd	Mean	Sd	Mean	Sd	Mean	Sd	Mean	Sd	Mean	Sd
Gender	Boys	3.70	2.04	6.85	1.76	1.58	0.69	3.42	1.23	3.26	1.26	14.71	4.91
	Girls	3.05	1.91	6.92	1.56	1.58	0.61	3.52	1.12	3.35	1.15	15.19	4.40
	*t*	7.23^***^		−0.94		−0.09		−1.97		−1.68		−2.27	
Sibling status	With siblings	3.47	2.06	6.85	1.64	1.57	0.66	3.46	1.20	3.29	1.20	15.08	4.56
	Without siblings	3.30	1.97	6.90	1.65	1.58	0.64	3.48	1.64	3.32	1.20	14.96	4.65
	*t*	1.48		−0.64		−0.22		−0.30		−0.47		0.44	
Grade	Freshman	3.63	1.98	7.08	1.55	1.46	0.59	3.41	1.08	3.24	1.10	14.52	4.52
	Sophomore	2.93	1.90	6.72	1.69	1.68	0.68	3.58	1.25	3.43	1.28	15.46	4.67
	Junior	4.67	1.92	6.98	1.81	1.54	0.60	3.06	1.03	2.89	1.08	14.39	4.65
	Senior	1.57	1.62	5.71	2.63	1.86	1.07	3.57	1.40	2.86	0.90	16.14	7.84
	*F*	41.57^***^		9.05^***^		18.93^***^		8.49^***^		9.59^***^		7.43^***^	

^***^p < 0.001.

### 3.3 Correlation analysis

As presented in Table 2, PA showed a significant positive association with psychological resilience. Conversely, it was negatively correlated with ST, DP, AN, and SMA. Psychological resilience demonstrated significant negative correlations with ST, DP, AN, and SMA. In addition, ST was positively related to DP, AN, and SMA. DP was strongly associated with AN and was also positively linked to SMA. Similarly, AN showed a significant positive relationship with SMA.

**Table 2:** Correlation analysis.

Variables	1	2	3	4	5	6
1 PA	–					
2 PR	0.134^***^	–				
3 ST	−0.068^**^	−0.192^***^	–			
4 DP	−0.117^***^	−0.267^***^	0.399^***^	–		
5 AN	−0.095^***^	−0.278^***^	0.410^***^	0.782^***^	–	
6 SMA	−0.130^***^	−0.209^***^	0.266^***^	0.362^***^	0.387^***^	–

^**^p < 0.01; ^***^p < 0.001.

### 3.4 Mediation model testing

**Figure 2:**
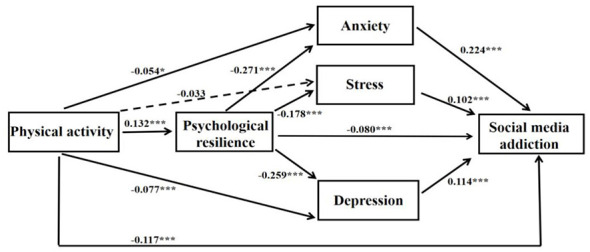
Chain mediation model.

After statistically controlling for demographic covariates, the mediation analysis results (see Table 3 and Figure 2) indicated that PA remained significantly negatively associated with SMA. Even after introducing psychological resilience, ST, DP, and AN into the model, the direct effect of PA on SMA persisted, suggesting a partial mediation effect. Furthermore, PA was positively related to psychological resilience, while it was negatively associated with DP, and AN. Psychological resilience was found to significantly reduce levels of ST, DP, and AN. Furthermore, psychological resilience was negatively correlated with social AN disorder SMA. Finally, negative emotional states were positively associated with SMA, including ST, DP, and AN.

**Table 3:** Mediation model test.

Outcome variables	Predictor variables	β	SE	*t*	R^2^	*F*
SMA	PA	−0.117	0.022	−5.251^***^	0.026	7.800^***^
PR	PA	0.132	0.023	5.935^***^	0.039	11.851^***^
ST	PA	−0.033	0.022	−1.490	0.052	13.884^***^
	PR	−0.178	0.022	−8.041^***^		
DP	PA	−0.077	0.022	−3.534^***^	0.080	21.923^***^
	PR	−0.259	0.022	−11.903^***^		
AN	PA	−0.054	0.022	−2.452^*^	0.083	22.801^***^
	PR	−0.271	0.022	−12.502^***^		
SMA	PA	−0.068	0.021	−3.307^**^	0.188	42.573
	PR	−0.080	0.022	−3.728^***^		
	ST	0.102	0.022	4.547^***^		
	DP	0.114	0.033	3.490^***^		
	AN	0.224	0.034	6.831^***^		

^*^p < 0.05; ^**^p < 0.01; ^***^p < 0.001.

## 4 Discussion

This study examined the associations among PA, psychological resilience, AN, ST, DP, and SMA in university students, along with the mediating roles of psychological resilience and NE. The findings revealed that greater engagement in PA corresponded to lower levels of SMA. In addition, PA was linked to enhanced psychological resilience and reduced levels of ST, AN, and DP. Psychological resilience, in turn, corresponded to lower manifestations of negative emotional states. Among the emotional factors examined, ST and AN were more closely connected with SMA. Collectively, the results were consistent with the proposed hypotheses.

### 4.1 The impact of physical activity on social media addiction

A significant negative association between PA and SMA was observed, consistent with prior research (Poskotinova et al., 2021; Liu et al., 2024). From the perspective of behavioral structuring theory, regular activities are related to reduced impulsive and unplanned behaviors by shaping daily routines and behavioral organization (Baumeister and Vohs, 2007). PA typically involves structured schedules, goal-oriented engagement, and immediate feedback, all of which are associated with greater behavioral regulation. Such structured engagement is linked to a lower likelihood of excessive social media use during fragmented time periods (Sniehotta et al., 2005). Empirical research has also shown that students who regularly participate in PA report lower levels of impulsive media use and uncontrolled internet behaviors (Wang W. et al., 2025). SMA can be conceptualized as a maladaptive coping strategy comparable to substance-related behavioral dependence. Among university students, SMA is not necessarily rooted solely in external stressors or adverse life events but is closely related to variations in daily routines and self-regulatory capacity. When students achieve emotional regulation and psychological satisfaction through regular PA, reliance on social media as an alternative regulatory strategy appears reduced, which is associated with lower behavioral dependence. Compensatory gratification theory proposes that when individuals experience difficulty fulfilling expectations in real life, they may seek alternative channels for satisfaction. Within this framework, online social networking provides opportunities for attention and positive feedback from others, partially compensating for unmet needs in offline social interactions (Lu et al., 2020). This mechanism may be especially salient in the Chinese cultural setting, where collectivist norms and heightened parental expectations contribute to elevated stress among university students (Wang et al., 2017). Although the observed association between PA and SMA in this study was relatively modest (β = −0.117), this effect size is consistent with prior findings in social and health psychology, where behavioral outcomes are typically influenced by multiple interacting psychological, social, and environmental factors. Evidence from meta-analyses suggests that associations of this magnitude are common in behavioral research and should not be considered trivial (Weinerová et al., 2022; Cohen, 2013). Collectively, these findings support H1, demonstrating that greater engagement in PA corresponds to lower levels of SMA in university students.

### 4.2 Subgroup analysis of differences by academic year and gender

In addition, the present study found significant differences in physical activity and social media addiction across gender and academic grade levels. These findings are consistent with previous research, suggesting that males tend to report higher levels of physical activity participation, whereas females are more likely to exhibit greater emotional distress and a higher tendency toward problematic social media use (Zhihao et al., 2024; Wang et al., 2025a; Kusan, 2025). These differences may reflect broader variations in lifestyle patterns, academic demands, and psychosocial development among university students. Females are generally more sensitive to emotional experiences and are more likely to adopt emotion-focused coping strategies, which may increase their reliance on social media as a means of regulating negative emotions under stress (Wang Q. et al., 2025). In contrast, males are more likely to engage in behavioral coping strategies, such as PA, to manage stress, which may be associated with a reduced dependence on virtual environments (Zhihao et al., 2024). Therefore, gender differences are not only reflected at the behavioral level but may also be linked to distinct emotion regulation pathways that are associated with patterns of social media use. With regard to academic grade, students at different stages of university experience distinct developmental tasks and environmental pressures. Lower-grade students often face challenges related to adapting to university life and establishing interpersonal relationships, whereas higher-grade students tend to experience increased academic demands and employment-related stress. These factors may be associated with variations in both physical activity participation and reliance on social media (Compas, 1987). Taken together, these gender and grade differences provide important contextual insights into the associations between PA and SMA. They also suggest that intervention strategies may benefit from considering subgroup characteristics. For example, more structured opportunities for physical activity could be provided to groups with lower participation levels, while targeted guidance on media use and psychological adjustment may be particularly relevant for higher-risk groups.

### 4.3 The mediating role of psychological resilience

Our findings provide additional evidence regarding the mediating function of psychological resilience in the association between PA and SMA, further clarifying the psychological processes underlying this linkage. Moreover, these findings contribute to the understanding of potential intervention mechanisms. Although earlier studies have documented close connections between PA and psychological resilience, as well as between psychological resilience and SMA (Qiu et al., 2023; Zhao et al., 2022; Yam et al., 2024), our study extends this knowledge by situating these relationships within a Chinese university context and a mediation framework informed by the I-PACE model. In this context, intense academic competition, uniform evaluation standards, and high social expectations collectively shape students' stress experiences and coping strategies, making internal psychological resources particularly salient. These findings suggest that the protective effect of PA on SMA is not solely due to behavioral time displacement, but also operates indirectly by enhancing key psychological adaptive capacities. PR can thus be conceptualized as a critical protective factor that enables students to maintain psychological stability and adaptive functioning when facing stress, setbacks, and uncertainty—conditions commonly experienced during university life (Fletcher and Sarkar, 2013). Higher levels of PA were associated with greater PR, indicating that regular engagement in PA may foster the development of internal regulatory resources. This relationship can be understood through complementary mechanisms. Students with higher PA levels tend to exhibit stronger PR, which supports more stable emotional states when encountering academic demands, interpersonal conflicts, and future uncertainties. Regular PA may enhance PR by increasing self-efficacy, improving emotion regulation, and promoting adaptive cognitive appraisals (Bandura, 1997). Conversely, students with lower PR may lack effective coping strategies, increasing reliance on external, low-cost, and instant-feedback channels such as social media to manage negative emotions (Shiraly et al., 2024). Consistent with the I-PACE model, higher PR enables students to adopt constructive coping strategies in the face of stress or negative affect, rather than engaging in SMA for emotional avoidance or immediate compensation (Wu et al., 2024). Empirical evidence further supports this link: among university students, higher PR is associated with fewer Internet-related problems, including addictive behaviors, highlighting its role as a psychological protective factor (Robertson et al., 2018). Taken together, these cognitive and physiological processes help explain how PA promotes psychological resilience. Conversely, enhanced psychological resilience enables individuals to adopt more adaptive coping strategies, thereby reducing reliance on maladaptive behaviors such as excessive social media use for emotion regulation. Overall, the present findings demonstrate that psychological resilience is associated with the linkage between PA and SMA among university students (H2).

### 4.4 The mediating role of negative emotions

Based on the existing literature and our findings, close associations were identified among PA NE (including DP AN, and ST), and media addiction among university students (Che et al., 2025; Wang et al., 2025b; Wu et al., 2025), thereby supporting our initial hypotheses. University students who lack PA tend to remain in a prolonged state of low physical activity. This not only limits their opportunities to release ST and regulate emotions through exercise, but may also weaken the adaptive functioning of their physiological and psychological systems, thereby increasing the risk of internalizing problems such as DP, AN, and ST (Biddle and Asare, 2011). Students experiencing high levels of AN and ST are often more introverted and reluctant to engage in peer communication; however, they may turn to social media to escape or alleviate these negative emotional states, ultimately leading to excessive internet dependence (Liu et al., 2022). The allostatic load model explains that, in the long-term process of coping with academic, interpersonal, and environmental ST, individuals must continuously mobilize their physiological and psychological systems to maintain functional stability. When such regulation remains under high load for extended periods, allostatic load accumulates, thereby increasing the risk of internalizing problems such as AN, DP, and ST (McEwen, 1998; McEwen and Stellar, 1993). University students who lack PA often find it more difficult to effectively release daily ST. Their autonomic nervous and endocrine systems may remain in a state of chronic hyperactivation, making them more prone to emotional tension, increased fatigue, and reduced emotional recovery capacity, which in turn heightens vulnerability to AN and depressive symptoms (Juster et al., 2010). In contrast, regular PA is considered an important “allostatic load regulator,” as it helps individuals maintain a lower allostatic load by improving cardiovascular function, enhancing the flexibility of neuroendocrine systems, and reducing chronic stress-related physiological responses (Juster et al., 2011). Previous studies have shown that long-term perceived ST and emotional distress are significantly associated with more frequent and uncontrolled social media use. Individuals are more likely to temporarily relieve psychological tension through repeated checking and immersive use of social media (Elhai et al., 2017). Furthermore, under conditions of high psychological load, individuals tend to prioritize short-term emotional relief over long-term goal-oriented behavior when making behavioral choices. This decision-making tendency weakens self-regulation of social media use, facilitating a shift from instrumental use to habitual or even compulsive use patterns, ultimately increasing the risk of SMA (Hormes et al., 2014). In summary, our findings indicate that AN, DP, and ST mediate the relationship between PA and SMA among university students (H3).

### 4.5 The chain-mediating effect of psychological resilience and negative emotions among college students

In addition, the present study identified negative correlations between PR and DP, AN, and ST. PR is widely recognized as a core psychological resource that supports emotional stability and adaptive functioning under ST and adversity. It is closely linked to individuals' cognitive appraisal processes and emotional response patterns. Emotion regulation theory holds that responses to stress-related events depend substantially on the regulation strategies individuals employ. Higher levels of PR are associated with greater use of adaptive strategies such as cognitive reappraisal and problem-focused coping, which are linked to reduced emotional distress (Gross, 2015). In contrast, lower PR is associated with maladaptive appraisal patterns, heightened threat perception, and more intense negative emotional reactions (Tugade and Fredrickson, 2004). From a neurobiological perspective, PR has been associated with functional connectivity between the prefrontal cortex and limbic regions. The prefrontal cortex is involved in emotion regulation and ST inhibition, whereas limbic structures such as the amygdala are implicated in threat detection and negative affect processing. Individuals with higher PR exhibit stronger top-down regulatory control of the prefrontal cortex over limbic activation (Waugh et al., 2011), whereas lower resilience is associated with amygdala hyperactivation and weaker prefrontal modulation under ST exposure (Feder et al., 2009). PR is also associated with ST recovery capacity. Individuals with lower resilience tend to recover more slowly from stress-related events, which is linked to elevated risks of chronic ST and emotional disorders (Southwick et al., 2014). Overall, the findings demonstrate that PR is negatively associated with DP, AN, and ST (H4). Furthermore, AN, ST, and DP are linked to the association between PA and SMA among university students, supporting the final hypothesis. This biological explanation constitutes a key psychological vulnerability factor, rather than a biological susceptibility.

### 4.6 Differential mediating roles of psychological resilience and negative emotion

The mediation model results (Table 3 and Figure 2) provide evidence for a partial mediating mechanism underlying the association between PA and SMA, suggesting that PA is linked to SMA through multiple psychological pathways. Although the direct association remains significant (β = −0.117, *p* < 0.001), the substantial total indirect effect (41.88%) indicates that this relationship is largely accounted for by underlying psychological processes. A key contribution of the present study lies in distinguishing the functionally distinct roles of psychological resilience (PR) and negative emotional factors (DP, AN, and ST) within this framework. Specifically, PR appears to operate as a resource-based protective pathway, whereas DP, AN, and ST reflect symptom-based risk pathways, highlighting two fundamentally different processes through which PA is associated with SMA. From a protective perspective, PR represents an internal adaptive resource that supports self-regulation and effective coping with stress. The results show that PA is positively associated with PR (β = 0.132, *p* < 0.001), and higher PR is linked to lower levels of SMA (β = −0.080, *p* < 0.001). This pathway suggests that PA is not only associated with reduced problematic behavior directly, but is also related to the strengthening of psychological resources that enable individuals to adopt more adaptive coping strategies under stress. In this sense, PR may function as a buffering factor, reducing the likelihood that individuals rely on SMA as a compensatory or avoidant coping strategy. In contrast, negative emotional factors reflect a reactive vulnerability pathway. The findings indicate that PA is associated with lower levels of DP, AN, and ST, while these variables are positively related to SMA. Among them, AN shows the strongest association with SMA (β = 0.224), followed by DP and ST, suggesting that heightened emotional arousal and tension may be particularly relevant to problematic engagement with social media. This pattern implies that individuals experiencing elevated negative emotions may be more likely to turn to social media for immediate emotional relief, distraction, or avoidance, thereby increasing the likelihood of SMA.

Importantly, the relative contributions of these mediators further clarify their roles. Bootstrap analyses (Table 4) indicate that AN accounts for the largest proportion of the total effect (10.26%), followed by PR, DP and ST. These findings suggest that, although multiple emotional pathways are involved, AN may represent a more proximal and salient emotional correlate of SMA, while PR reflects a more stable and enduring protective factor.

**Table 4:** Mediation model path analysis.

Intermediate path	Effect size	SE	Bootstrap 95% CI	Proportion of mediating effect
Total effect	−0.117	0.022	−0.161, −0.073	
Direct effect	−0.068	0.021	−0.109, −0.028	
Total indirect effect	−0.049	0.010	−0.070, −0.003	41.88%
PA → AN → SMA	−0.012	0.005	−0.023, −0.002	10.26%
PA → PR → SMA	−0.010	0.004	−0.019, −0.003	8.55%
PA → ST → SMA	−0.003	0.003	−0.009, 0.001	2.56%
PA → DP → SMA	−0.009	0.004	−0.017, −0.002	7.69%
PA → PR → AN → SMA	−0.008	0.002	−0.012, −0.004	6.84%
PA → PR → ST → SMA	−0.002	0.001	−0.004, −0.001	1.71%
PA → PR → DP → SMA	−0.004	0.001	−0.007, −0.001	3.42%

Taken together, these findings highlight a dual-pathway mechanism, in which PA is associated with both enhanced internal psychological resources (PR) and reduced emotional vulnerability (particularly AN), thereby relating to lower levels of SMA. This integrated framework provides a more comprehensive account than models focusing on a single mediator and underscores the importance of considering both positive and negative psychological processes in understanding problematic social media use.

This study systematically examined the interconnections among PA, PR, NE (AN, DP, and ST), and SMA in university students. By constructing and validating a chain mediation framework centered on PR and negative emotional factors, the study extends theoretical explanations of how health-related behaviors are linked to problematic media use. The findings showed that PA not only suppresses SMA through direct behavioral substitution but, more importantly, enhances individuals' PR, which in turn reduces internalizing problems such as AN, DP, and ST, ultimately decreasing reliance on social media as an emotion regulation tool. This result deepens our understanding of how positive lifestyle behaviors influence addictive behaviors through psychological adaptation mechanisms and highlights the pivotal bridging role of PR in the PA–SMA relationship. University students are at a critical stage of psychological development and social adaptation, during which academic ST, interpersonal challenges, and future uncertainty often converge, making them particularly vulnerable to NE and problematic media use. By integrating PR and NE within a unified analytical framework, the present study demonstrates that the association between positive health behaviors and problematic behaviors reflects a multi-layered psychological structure rather than a simple linear pattern. These findings contribute to a more comprehensive understanding of the psychological foundations of SMA among contemporary university students and offer a basis for examining similar mechanisms across different cultural and developmental contexts. The study also has practical implications. Interventions targeting SMA among university students may benefit from emphasizing enhancement of PR through PA, alongside the alleviation of AN, DP, and ST. Universities may incorporate regular PA into mental health promotion initiatives and student development programs, integrating it with resilience-building and emotion management strategies to address SMA. From a practical perspective, these distinct pathways suggest that intervention strategies should adopt a multidimensional approach. While promoting PA may serve as a foundational strategy, greater emphasis should also be placed on strengthening PR, for example through coping skills training. In addition, reducing AN through targeted mental health support programs appears particularly important. Interventions that simultaneously enhance protective psychological resources and reduce emotional risk factors may be more effective in mitigating SMA among university students. Future research may further adopt longitudinal or experimental designs to examine the long-term effects of different types, intensities, and durations of PA on PR and SMA, thereby advancing more precise and sustainable intervention practices.

## 5 Research limitations and future directions

This study has several limitations that warrant acknowledgment. First, its cross-sectional design precludes causal inferences. Second, the sample, restricted to Chinese university students, limits generalizability and external validity. Third, reliance on self-report questionnaires, despite using validated instruments, may introduce social desirability bias and subjective errors. Fourth, the lack of differentiation in PA type, frequency, or duration overlooks potential heterogeneous effects on psychological states and SMA, highlighting the need for more refined PA classifications. Fifth, although the sample covered multiple geographical regions within China, it may not fully represent the diversity of the national university student population. Variations in socioeconomic status, urban–rural distribution, and educational resources may not have been sufficiently captured, potentially limiting the heterogeneity of the sample. This may lead to higher within-group similarity relative to between-group differences, reflecting potential intra-cluster correlation, which could reduce estimation precision and affect the statistical independence and external validity of the findings. In addition, the cultural context should be taken into consideration when interpreting the results. The present study was conducted within a Chinese sociocultural setting, where social norms, family structures, and patterns of social media use may differ from those in Western or other cultural contexts. These cultural differences may influence both psychological processes (e.g., resilience and emotional regulation) and behavioral patterns related to SMA. Therefore, caution is warranted when generalizing the findings to populations from different cultural backgrounds. Future research could further advance this line of inquiry in several important ways. First, more nuanced investigations are needed to examine the differential effects of various types of PA, such as individual vs. group-based activities, as well as differences in frequency, intensity, and duration. These distinctions may help clarify how specific forms of engagement influence PR, emotional states, and SMA. Second, future studies could compare students from different universities or educational contexts to explore how environmental and institutional factors shape behavioral patterns. Differences in campus culture, academic pressure, and access to recreational resources may contribute to variations in PA and social media use. Third, longitudinal and experimental study designs, as well as latent variable methods (such as SEM), are needed to compensate for the limitations of cross-sectional data and further improve measurement accuracy and model robustness. Longitudinal studies tracking students over time would help clarify the temporal ordering among PA, PR, NE, and SMA. Experimental or intervention-based studies, such as structured physical activity programs implemented in university settings, could further test causal mechanisms and evaluate whether increasing PA leads to improvements in psychological outcomes and reductions in problematic social media use. In addition, future research could incorporate objective measurement approaches (e.g., wearable fitness trackers and physiological indicators) to complement self-report data and enhance measurement precision. Finally, these findings provide a basis for developing targeted intervention strategies. Universities may consider integrating physical activity programs with mental health initiatives, such as resilience training and stress management workshops, to address both behavioral and psychological pathways underlying SMA.

## 6 Conclusion

This study systematically examined the relationships among PA, SMA, psychological resilience, and NE (AN, DP, and ST) in university students, constructing a multiple mediation model to clarify the psychological mechanisms by which PA influences SMA. Results indicate that PA is significantly associated with SMA levels, and its effects are mediated through changes in PR and NE—underscoring the central role of emotion regulation and psychological adaptation. These findings extend prior research, which has largely framed SMA in terms of behavioral frequency or usage duration, by highlighting how positive lifestyle behaviors shape psychological functioning and behavioral patterns. From a practical standpoint, future interventions should integrate quantifiable psychological indicators and stratified assessments of students' physical activity levels, emotional states, and addiction risk. Such an approach would provide a scientific foundation for developing more targeted, actionable prevention and intervention strategies.

## Data Availability

The raw data supporting the conclusions of this article will be made available by the authors, without undue reservation.
